# Co‐Authoring and Reporting on Lived Experience Engagement in Mental Health and/or Substance Research: A Qualitative Study and Guidance Document

**DOI:** 10.1111/hex.70198

**Published:** 2025-03-12

**Authors:** Natasha Y. Sheikhan, Kerry Kuluski, Melissa Hiebert, Charlotte Munro, Vivien Cappe, Mary Rose van Kesteren, Sean Kidd, Lisa D. Hawke

**Affiliations:** ^1^ Institute of Health Policy, Management, and Evaluation University of Toronto Toronto Canada; ^2^ Centre for Addiction and Mental Health Toronto Canada; ^3^ Institute for Better Health, Trillium Health Partners Mississauga Canada; ^4^ Department of Psychiatry University of Toronto Toronto Canada

**Keywords:** family engagement, guideline, mental health, patient and public involvement, patient engagement, people with lived experience, qualitative research, substance use

## Abstract

**Introduction:**

There is a move towards engaging people with lived experience and families (PWLE/F)—also referred to as PWLE/F engagement—in mental health and/or substance use research. However, PWLE/F engagement is inadequately reported on in mental health and/or substance use research papers.

**Objective:**

To understand what PWLE/F and researchers perceive are important components to report on related to engagement in mental health and/or substance use research.

**Methods:**

This study included a qualitative description study underpinned by pragmatism. Data were collected through virtual interviews with 13 PWLE/F and 12 researchers across Canada and analysed using template analysis. The results were used to develop a reporting guidance document for engagement in mental health and/or substance use research.

**Results:**

The results from the template analysis were structured through the following themes: (1) establishing the need for a guidance document; (2) aspects of engagement to report and reflect on; (3) guidance around co‐authorship with PWLE/F; (4) practical tips for reporting on engagement and (5) considerations for journals. Participants identified a need for tailored guidance that is flexible and reflective, yet can promote transparency, accountability and learning in the field. A reporting guidance document was developed for engagement in mental health and/or substance use research that balances flexibility and standardisation while incorporating reflection into reporting. Guidance around co‐authorship with PWLE/F partners was also included.

**Conclusion:**

The guidance document is intended to be used as a roadmap to help guide authors to meaningfully write about engagement without the rigid boundaries of a reporting guideline. We encourage research teams that engage PWLE/F in mental health and/or substance use research to consider using the guidance document as they write up their work.

**Patient and Public Involvement:**

PWLE/F members were engaged throughout the study from conception to manuscript production. This included a PWLE partner on the doctoral committee and a Lived Experience Advisory Group consisting of two PWLE and one family partner.

## 1 Introduction

Engaging people with lived experience and their families (PWLE/F) as collaborators throughout the research process is increasingly recognised as a best practice in health research [1]. Meaningful PWLE/F engagement—also referred to as patient engagement, patient and public involvement and service user involvement—is defined as planning, supporting and valuing PWLE/F partners throughout the research process and within a positive research environment [2]. PWLE/F engagement is especially relevant to mental health and/or substance use research given the historical and persistent oppressive practices in psychiatry, fuelling a growing body of research in the field [3]. Current research shows that engagement has a positive impact on mental health and/or substance use research, improving aspects of the research process such as recruitment, methods and relevance of the results [4, 5, 6]. However, engagement is inconsistently reported on in mental health and/or substance use research [3, 6].

In reviews that explored the topic of engagement in mental health and/or substance use, several have described inconsistent reporting of engagement in research studies, such as limited information on the depth of engagement [7], contextual factors like sociodemographic characteristics [3, 8], terminologies used to define engagement and PWLE/F [3] and co‐authorship with PWLE/F [9]. Limited reporting has made it difficult to evaluate the quality impact of engagement in such studies [6, 7, 10, 11]. As clear reporting can help us understand, learn and appraise engagement activities, efforts should be made to ensure meaningful reporting practices in mental health and/or substance use research.

Reporting guidelines, for instance, could potentially be helpful to ensure transparent and consistent reporting of engagement [12]. Indeed, there are evidence‐based reporting guidelines such as the Guidance for Reporting Involvement of Patients and the Public (GRIPP) and GRIPP2 checklists that can be used to improve the reporting of engagement methods, results and impact in health and social care research [13]. There are two forms of GRIPP2: the long form (GRIPP2‐LF) and the short form (GRIPP2‐SF) [13]. In a study where PWLE/F co‐researchers used both the GRIPP2‐LF and GRIPP2‐SF, the short form was found to be useful and straightforward, while the long form was found to be complicated and not user‐friendly [14]. However, the GRIPP2 has been critiqued as being limited in terms of reflexive reporting and promoting a false sense of objectivity [15]. Moreover, although the GRIPP2 can be effective in improving the reporting of engagement in health research, it may overlook several dimensions important to mental health and/or substance use research, such as facets of power dynamics unique to the context of mental health and/or substance use and considerations around co‐authorship and disclosure [16].

To our knowledge, there is currently only one reporting framework for engagement that is illness‐specific—the framework by Hamilton et al. [17]—to advance the reporting of engagement in rheumatology research. However, there are specific considerations to mental health and/or substance use research, such as stigma and power imbalances, that are not captured in the framework, underscoring the need for illness‐specific reporting frameworks that reflect the unique considerations of engagement in different fields. This would ensure that reporting is aligned with the lived experiences of those engaged in the research.

There are currently no reporting tools or guidance specific to engagement in mental health and/or substance use research. This is a missed opportunity for building a tool rooted in the unique experiences of PWLE/F related to mental health and/or substance use—especially as the current evidence state shows inconsistent reporting [3, 8]. As suggested in scoping reviews by Sheikhan et al. [3] and Hawke et al. [18], it is important to explore the lack of consistency when reporting on engagement within mental health and/or substance use research. Therefore, understanding the need for reporting guidance tailored to mental health and/or substance use research and developing a reporting guidance document rooted in the experiences of PWLE/F and researchers are critical. We therefore sought to provide a rich description of what PWLE/F partners and researchers perceive are valuable components to report on regarding engagement in mental health and/or substance use research.

## 2 Methods

### 2.1 Design

A qualitative descriptive approach was used to understand expert perspectives regarding reporting on engagement in mental health and/or substance use research. Qualitative description is appropriate for this study as it can provide a first‐hand description of the phenomenon under investigation from the perspectives of those directly involved—PWLE/F and researchers [19]. As the findings were used to develop a reporting guidance document, a qualitative descriptive approach provided rich descriptions in straightforward language. A pragmatist epistemology underpinned this study as it aligns well with qualitative description and patient engagement research [20]. Pragmatism also focuses on real‐world practice, which is ideal given that we intended to develop a practical guidance document that can be applied in research settings. The Big Q Qualitative Reporting Guidelines (BQQRG) were followed to guide qualitative reporting [21].

### 2.2 Context

The Canada‐wide study took place virtually via the Zoom teleconferencing system. The study is part of a three‐paper PhD dissertation conducted based out of the University of Toronto and Centre of Addiction and Mental Health. The research team consists of PWLE/F and researchers, including the doctoral candidate who identifies as a PWLE and has been engaged as a PWLE partner in health systems work, their co‐supervisors who both conduct PWLE/F engagement research, the members of the doctoral committee (including a PWLE partner) and a Lived Experience Advisory Group with three members. Details on team members and the engagement dynamics are described in Table 1. Given the context and the ‘insider’ social positioning, reflexive journaling was used by the first author as part of research practice.

**Table 1:** Experiences of PWLE/F engagement during the research process.

Areas to report	PWLE partner on PhD Committee	PWLE/F advisory group
Who was involved?	The PhD Committee consisted of the co‐supervisors (L.D.H. and K.K.) and two committee members (S.K. and M.H.), including one PWLE partner (M.H.) who identified as having lived experience related to mental health. M.H. is a research engagement coordinator, with the role of developing and implementing a research engagement programme within a mental health institution. Through their role, they have experience supporting PWLE/F with the co‐authorship process.	Advisory group members consisted of a person who identified as having lived experience related to mental health and/or substance use (C.M.), a person who identified as having family experience related to mental health and/or substance use (V.C.), and a person who identified as having both lived and family experience related to mental health and/or substance use (M.V.K.). All advisors also had experience being engaged on a research team and as co‐authors on a peer‐reviewed publication.
*Note: In addition to the PWLE/F described, the first author (N.Y.S.) also identifies as a PWLE and has been engaged as a patient partner in several health systems roles.		
What were the activities, roles and responsibilities?	M.H. was engaged as a PhD committee member with equal decision‐making power. Activities included biweekly meetings during the writing process to brainstorm ideas, discuss and reflect on the analysis and make key decisions about the paper's direction. Roles and responsibilities as a committee member included supporting the PhD proposal development, approving the PhD proposal and progress reports, attending committee meetings, working collaboratively as a co‐author on the dissertation papers and participating in decision‐making as a voting committee member. M.H. also supported the data collection and recruitment of both study participants and members of the PWLE/F Advisory Group. Support and mentorship were available from the research team.	Advisory group members attended and participated in regular meetings via Zoom throughout the project. Activities included providing feedback during the meetings, reviewing drafts and providing feedback offline, brainstorming ideas, discussing and reflecting on the analysis and making key decisions about the paper's direction as co‐authors. The advisors also contributed to the recruitment plan and shared the study flyers, refined the interview guide and tested the interview questions. N.Y.S. provided informal training for the advisors on qualitative coding before the analysis stage and held sessions to clarify the research process and language.
How did you go about the engagement process?	M.H. was recruited through CAMH by the PhD co‐supervisor (L.D.H.). To become an official committee member, we underwent a formalised process to receive a thesis‐only appointment from the university. N.Y.S., L.D.H. and K.K. advocated for having a PWLE partner as a committee member and navigated through institutional processes to equalise power and formally recognise the role. *Challenges*. There were, however, barriers at the institutional level. There was initial resistance from the department to formally have a PWLE partner as a PhD committee member as they were not a ‘faculty member’, reflecting academia's exclusionary culture and lack of formal processes for recognising the value of non‐academic/lived experience contributions. This required us to find loopholes and challenge, rather than accept, the status quo. As a team, we ensured the environment was inclusive and that M.H. was treated as an equal member with voting rights rather than an outsider on the committee. We were all committed to changing the culture and creating new norms for engagement in PhD work. *Facilitators*. All team members recognised the value of engagement and were motivated to meaningfully do it despite the initial institutional challenges. Both supervisors were highly supportive throughout the process. Moreover, institutional buy‐in from CAMH facilitated the process; support from the programme director at CAMH was essential to allow M.H. to embed the time spent as a committee member into their CAMH role. Lastly, structuring decision‐making processes to share power equally among committee members facilitated an inclusive space and directly diffused power imbalances. *Context and Compensation*. Engagement was mostly virtual, with occasional in‐person events. Honorarium was discussed but not provided as the M.H.'s contributions became integrated into their job as an engagement specialist at CAMH. *Relationships*. Strong relationships were built through regular check‐ins and meetings outside of the committee meetings, going beyond standard PhD committee practices. M.H. and N.Y.S. both made reciprocal efforts to build a relationship, foster trust and produce research that is meaningfully rooted in lived experience voices.	Advisory group members were recruited through CAMH by the PWLE partner on the PhD committee (M.H.) and the co‐supervisor (L.D.H.). Meetings occurred virtually, with one in‐person event to present the work at a conference. *Facilitators*. The engagement process was facilitated through open communication with N.Y.S., such as offline meetings and emails always being available. The advisors felt that N.Y.S. was flexible, listened actively, was good with communication (e.g., always replied to emails) and created a space where the advisors did not hesitate to reach out or felt they were bothering anyone. N.Y.S. checked in with advisors to make sure they could attend the meetings and was accommodating when the advisors could not attend (e.g., rescheduling meetings or having separate one‐on‐ones to ensure everyone was heard). The advisors did not experience any barriers because we were respectful and treated each other as equals—everyone was open and receptive to feedback and actively participated in the meetings. N.Y.S. created a safe and productive environment that allowed for better insights, where people felt comfortable being their authentic selves and fostered a feeling of belonging. *Equal partnership*. NYS and the advisors made sure decisions were shared—when ideas were presented and there were concerns, we worked on them as a team, talked through new solutions and only moved on when there was agreeance amongst the team. For example, during paper edits, N.Y.S. clarified why something was not going to get changed; this was proactively explained so the advisors did not feel ignored and their concerns were addressed. Advisory group members felt like equals on the team and in decision‐making and felt confident asking questions. *Relationships*. Strong relationships were built throughout the process. N.Y.S. held one‐on‐one meetings with each advisor before the first advisory group meeting to establish rapport. The meetings were not one‐sided, and N.Y.S. shared about her own lived experience, which fostered trust. Advisors felt supported throughout the process. Having a strong relationship had a positive impact on the quality of the work produced, and everyone felt comfortable sharing and learning. *Compensation and Accommodations*. Advisors were compensated for their time. N.Y.S. accommodate compensation requests (e.g., flexibility around gift cards vs. e‐transfers). Other accommodations included removing the password on Zoom for accessibility, providing materials in advance, contacting members for meeting reminders, and being flexible about meetings.
When did engagement occur?	Engagement occurred since the inception of the PhD committee for a 2‐year period. During the present paper, M.H. and N.Y.S. had bi‐weekly meetings. Other meetings included the PhD committee meetings, the proposal defence and the incoming PhD defence.	Engagement occurred for a year, starting January 2024 till January 2025. Meetings ranged from 1 to 3 times a month, with flexibility to meet as needed.
What was the impact?	*Impact on research*. Engagement on the PhD committee improved data collection, data analysis, the relevance of findings, the guidance document and paper drafts. Engagement also guided the PhD thesis at a higher level, including improvements to the PhD proposal, facilitating the engagement of the advisors (e.g., recruitment of advisors and planning content for meetings) and helping reflect and apply the practical aspects of engagement. *Impact on partner*. M.H. expressed that being on the PhD committee helped improve their confidence and self‐esteem—their lived and professional expertise was valued, even though they do not hold a PhD. They also found it valuable for their professional growth and felt being a part of the project gave them greater awareness of scholarship in engagement, which will, in turn, increase the quality of the engagement support offered at their institution. *Impact on researchers*. Having a PWLE partner on the PhD committee had a positive impact on N.Y.S's academic journey and helped guide their PhD. N.Y.S. felt M.H. provided peer support, improved their PhD (including increasing the relevance of PhD outputs to PWLE/F) and better prepared them for their PhD defence.	*Impact on research*. Engagement with the advisory group members improved data collection (e.g., recruitment and interview guide), added rigour and reflexivity to the data analysis and increased the relevance of the findings. Moreover, engagement improved the guidance document (e.g., made the language and content more relevant) and improved the paper drafts. *Impact on advisors*. Advisors expressed that being a part of the research process had a major impact on their confidence, expanded their critical thinking skills and helped them approach situations from different angles and positionality. Advisors felt like it was a great learning experience. Two advisors shared that this helped with feelings of isolation and improved their mental health because of the connection we built together. Advisors felt proud of their accomplishments during the project, such as participating in conferences, poster presentations and publications. Overall, they felt fortunate to be involved and that their experience on the project created more opportunities for them. *Impact on researchers*. Engaging with the advisory group had a major impact on the PhD candidate (N.Y.S.). Similar to the advisors, N.Y.S. felt it was a great learning experience that built her critical thinking and facilitation skills, deepened her understanding of the PhD topic and helped her understand lived experiences that were different than her own. Advisory group members and N.Y.S. felt it validated the value of engagement and contributed to the engagement movement.

*Note:* This table was completed together with the first author and PWLE/F partners.

### 2.3 Participants

The study included both PWLE/F partners and researchers over 18 years old who resided in Canada. Specifically, PWLE/F had to identify as having personal or family experience related to mental health and/or substance use, have been engaged as a PWLE/F partner in research within the last 3 years and self‐reported having contributed to a journal article as a co‐author or as an acknowledgement. Researchers had to conduct research related to mental health or substance use and have experience engaging PWLE/F in research and reporting on engagement research within the last 3 years.

To recruit a diverse sample, study flyers were circulated via email and social media through our personal and professional networks using purposive and snowball sampling techniques, in addition to contacting co‐authors based in Canada on relevant peer‐reviewed publications. The dataset size was determined based on information power and pragmatism [22, 23]; considerations included the narrow aim and specificity of the research question, the dialogue strength and feasibility. Recruitment started in April 2024 and ended in August 2024. The final sample included 13 PWLE/F and 12 researchers.

### 2.4 Procedure

Data were collected using semi‐structured, one‐on‐one interviews with PWLE/F and researchers. Guided by pragmatism, semi‐structured interviews were used to gain an in‐depth understanding of participants' perspectives on the phenomenon of reporting [24]. Interviews were conducted online via Zoom and ranged from 25 to 80 min long, averaging 51.4 min. The interview guide was designed based on findings from a scoping review by Sheikhan et al., [3] the Patient Engagement in Research framework by Hamilton et al. [2] and ongoing feedback from members of the doctoral committee and Lived Experience Advisory Group. Specifically, the advisory group helped refine the interview guide questions to ensure they were relevant to the participants. Participants were asked questions related to their experience with reporting on engagement, their perceptions regarding the utility of a reporting guideline and what components they perceived are important to report on. The interview guide was pilot‐tested with researchers and PWLE/F partners. An Information and Consent form was circulated to participants who expressed interest in the study through email. Written informed consent was obtained for all participants via the REDCap software before the interview. All interviews were audio‐recorded on Zoom, transcribed, anonymized and then uploaded to NVivo 14 for analysis. Research Ethics Board approval was received from the University of Toronto.

### 2.5 Data Analysis

Template analysis, a form of thematic analysis that involves hierarchical coding, was used to analyse the data [25]. Template analysis is a flexible analytic approach that involves developing a coding template based on a subset of the data and subsequently applying and revising the template to further data [25]. Template analysis was chosen as the analytic approach due to the flexible coding structure and alignment with pragmatic epistemology. Moreover, template analysis aligns well with a collaborative team analysis, where teams can work together throughout the analysis and template development—this was ideal given the extensive PWLE/F engagement during analysis [26]. A mix of *top‐down* (data generated from a priori themes) and *bottom‐up* (themes emerging from the data) approaches to analysis were used as we had defined a theme beforehand around areas to report on in engagement; however, the rest of the themes and sub‐themes were grounded in the data.

Coding was an iterative process conducted primarily by the first author, with codes and preliminary themes being brought to the doctoral committee members and Lived Experience Advisory Group throughout the analysis to enhance trustworthiness. In collaboration with PWLE/F, themes and sub‐themes were used to draft key dimensions and items for a reporting guidance document specific to engagement in mental health and/or substance use research. Template analysis was therefore useful as it allowed us to meaningfully and efficiently integrate the qualitative findings to develop a document rooted in PWLE/F and researcher views.

### 2.6 PWLE/F Engagement

PWLE/F engagement is described in Table 1. The guidance document in Appendix 1 was used to guide the reporting of engagement in this study.

## 3 Results

Participant characteristics are shown in Table 2. A total of 13 PWLE/F partners and 12 researchers across Canada were included in this study. Approximately half of the participants resided in the province of Ontario, the largest province in Canada. Age ranged from 24 to 63, with an average of 39.5. For ethnicity, 40% of participants identified as being racialized and 60% as non‐racialized. The results from the template analysis are structured through the following themes: (1) establishing the need for a guidance document; (2) areas to report and reflect on engagement; (3) guidance around co‐authorship with PWLE/F; (4) practical tips for reporting on engagement; and (5) considerations for journals. These results were used to create the guidance document for reporting on engagement in mental health and/or substance use research (Appendix 1). Representative quotes are identified with a participant number, then with a P for PWLE/F and with an R for researchers.

**Table 2:** Participant characteristics.

**Demographic**	*N*
Participant group	
PWLE partner/advisor	9
Family partner/advisor*	4
Researcher	12
Length of experience with engagement in mental health and substance use research	
1–5 years	14
6+ years	11
Location in Canada	
Ontario	13
British Columbia	8
Alberta	2
Quebec	1
Saskatchewan	1
Age	
Range	24–63
Average	39.5
Gender	
Man	8
Woman	15
Genderqueer or non‐binary	2
Ethnicity	
Racialized	10
Non‐racialized	15

* Three of the four family partners also identified as being a PWLE.

### 3.1 Establishing the Need for a Guidance Document

As participants reflected on their experiences with reporting, they established a need for a guidance document specific to reporting on engagement in mental health and/or substance use research. Key considerations for the guidance document included a need for guidance to improve standards and transparency, differentiating the guidance from other guidelines and ensuring resources are available if engaging with Indigenous communities.

#### 3.1.1 Need for Guidance to Promote Transparency and Accountability

Most participants highlighted a general need for guidance related to reporting on engagement in mental health and/or substance use research. Participants saw a guidance document as a way to address the limitations of scant reporting and improve transparency and accountability in the field. Many felt that providing more detail on engagement would help others learn, such as for teams looking to ‘replicate a similar paper’ (P01‐P). As such, reporting guidance was viewed as a helpful resource for research teams new to engagement.

A few researchers felt that reporting guidance would have been useful when they were new to engagement research, especially as ‘there doesn't seem to be one gold standard right now in terms of the level of detail to provide’ (P02‐R). As both researchers and PWLE/F struggled with what to include in peer‐reviewed papers, many felt that a guidance document could provide clarity and standardise how authors report on engagement. Several felt standardisation was important for improving consistency, rigour and trustworthiness. A few participants considered standardised reporting as a way of evaluating or critically appraising engagement activities. For instance:

‘If there's no standard reporting, we can't really evaluate how good or how authentic the patient engagement was.’ (P03‐R)

‘…on the critical appraisal side, it gives people something to think about as well and better understand and situate how findings came to be.’ (P04‐P)

Some believed that a guidance document would be ‘especially important in the mental health and addictions world’ (P05‐R), given the prominent power asymmetries in the field. In contrast, another researcher stated, ‘I'd use it across all my engagement work, not just in mental health’ (P06‐R), suggesting broader applicability to other areas of research. Despite differing views, the overarching belief was that there are unique attributes in mental health and/or substance use research that garner the need for guidance tailored to the field.

#### 3.1.2 Differentiating Guidance From Other Guidelines

Participants emphasised the need to differentiate the guidance document from existing reporting guidelines. Many were critical of reporting guidelines; they felt that a guidance document for reporting on engagement in mental health and/or substance use research should not follow a checkbox approach or be too rigid:

‘I think guidance is a good idea, as long as it includes a disclaimer that this is inherently not a checkbox endeavour. If you're box‐ticking, you might be on the wrong track.’ (07‐R)

Instead, several participants valued flexibility. Participants perceived flexibility as allowing for greater reflection without disrupting the ‘natural flow of engagement’ (P08‐R). Researchers, in particular, felt that methodological flexibility was needed as reporting will differ for qualitative versus quantitative papers. Some described a major shortcoming as relying too much on guidelines; as one PWLE/F explained, ‘guidelines may limit people because some people don't see beyond the set of guidelines’ (P09‐P). Participants instead felt that the guidance document should be treated as considerations rather than being enforceable. For example:

‘No one wants anything to be mandatory. Like, that just feels like you're being forced into doing something even though you agree with it or don't agree with it. But if it's more organic and it allows for flexibility…I feel like that's better.’ (P10‐R)

Preferences around what the guidance document might look like varied; this included checklists, reflection points, guiding questions and a need for ‘clear guidelines in general around co‐authorship’ (P11‐P).

#### 3.1.3 Indigenous Considerations

As we interviewed participants across Canada, a few mentioned having specific guidance for Indigenous communities. They stressed that research teams should at least be acknowledging the absence of Indigenous perspectives:

‘As an Indigenous person reading these things, it's really validating to see at least the acknowledgement that like, hey, Indigenous considerations are not covered in this and that's wrong. It's a lacuna…It's a space that needs to be filled.’ (P12‐P)

Participants felt that having a reference to guidelines specific to engaging with Indigenous peoples in research would make the document more inclusive.

### 3.2 Aspects of Engagement to Report and Reflect on

PWLE/F and researchers described several areas of engagement they felt were important to report and reflect on. Six main areas—in the form of questions—were conceptualised: (1) *How* did you go about engagement? (2) *What* were the activities, roles and responsibilities? (3) *When* did engagement occur? (4) *Who* was involved? (5) *What* was the impact? and (6) *Why* did you engage?

#### 3.2.1 How Did You Go About the Engagement Process?

PWLE/F and researchers felt it was important for authors to describe how they went about engagement, including details on the engagement process, context and influencing factors. Some perceived it would be beneficial to explain how PWLE/F partners were recruited, any accommodations that were provided to make the space inclusive and the context in which the engagement took place (e.g., ‘Was it a virtual partnership? Was it in person?’ [P02‐R]). Compensation, in particular, was seen as a key facet to report on to normalise compensating PWLE/F partners in the literature; however, the exact amount of compensation provided was seen as unnecessary and potentially unethical to describe. Engagement barriers, facilitators and potential solutions or ‘lessons learned’ (P08‐R) were also mentioned as being relevant to report on:

‘How did you work with patient partners? Like was it a conflict situation? Did you address that conflict? Did you address that conflict early on? Did it become a big thing? Was it a little thing?’ (P09‐P)

Although some participants felt it might be difficult to discuss power imbalances in papers, many encouraged at least reflecting on power and potentially documenting that reflection:

‘How did you address power imbalances, which is sometimes a hard question to answer, but I think it's important to think about. How did you mitigate?’ (P13‐R)

#### 3.2.2 What Were the Activities, Roles and Responsibilities?

PWLE and researchers felt it would be useful to describe specific engagement activities, including the roles and responsibilities, to improve transparency. When describing activities, this could include describing the meetings, clarifying the specific roles of PWLE/F across research stages (e.g., ‘Are they analyzing?’ [P08‐R]) and describing the level of engagement. For example:

‘Acknowledging the role that they played as accurately as possible. So when you just say, “Oh, we had an advisory panel of people with lived experience.” Okay, so what did they do?’ (P14‐F)

A few participants felt it was critical to clarify ‘partner versus participant’ (P15‐F) in papers to prevent confusion regarding PWLE/F roles as research partners as opposed to research participants in the study. A few researchers also felt it was important to report on whether training was provided for the PWLE/F partners involved.

#### 3.2.3 When Did Engagement Occur?

In addition to reporting on the engagement process and activities, several participants felt it would be valuable to provide a description of the engagement timeline. For example:

‘It gives you an indication of the level of engagement. So, if you met bi‐weekly for like 4 months or 5 months or something like that, I think that would be important. Whereas if you just met one time, it just kind of shows the different levels of participation.’ (P13‐R)

Timeline details, such as the frequency of meetings, were seen as a way to understand the varying levels of engagement across studies.

#### 3.2.4 Who Was Involved?

Most PWLE/F and researchers felt it was necessary to provide some demographic background on the PWLE/F engaged. The level of detail, however, was seen as flexible and heavily dependent on how it might relate to the research question. For instance, one PWLE/F stated that ‘it just depends on the topic’ and that reporting on demographics ‘might be relevant if it's something that's very specific culturally or ethnically’ (P01‐P). When reflecting on describing PWLE/F in papers, some participants highlighted how there should be a fair balance between the descriptions of PWLE/F and researchers. For example:

‘If you do for people with lived experience, you better do it for everyone. I would say otherwise it's pretty discriminatory.’ (P16‐P)

#### 3.2.5 What Was the Impact?

Most participants considered the impact of engagement as a key aspect to report on for increased transparency and to help others learn. This included the impact of engagement on PWLE/F, researchers and the research itself. Some felt that impact was more relevant to report on in a process or methods paper. When deciding whether to write about impact, participants felt it was ‘case by case dependent’ (P13‐R). However, some highlighted how impact may be ‘hard to judge’ (P01‐P) and difficult to report on given its subjective nature.

Several participants felt it was necessary to report on how impact was measured or evaluated. One researcher stated, ‘if you're telling us that youth researchers said this was a positive experience, how did you gain that information?’ (P06‐R). This related to concerns around how reporting on impact should be a collective decision made with PWLE/F—rather than only researchers—to ensure the narrative is authentic. For example:

‘If everyone on the team feels like there's value in reporting on the impact of engagement and also that it can be done in an authentic fashion that does not generate some twisted story about how things went—then great, there's immense value in that.’ (P04‐P)

Participants had mixed opinions about reporting on negative impacts. A few felt it was not necessary to report on negative or no impacts, while others felt it could strengthen the evidence base:

‘I think people should write about negative impacts. So it's more realistic for people who are getting into it because it can be done wrong in many ways.’ (P17‐P)

#### 3.2.6 Additional Reflection Points

Some participants highlighted areas to reflect on, such as co‐authorship, impact, power, positionality and the engagement process. These areas were seen as not necessarily critical to include in papers, yet still important for reflection. Several PWLE/F participants felt researchers should reflect on co‐authorship. Power and positionality were identified by both PWLE/F and researchers as important facets of engagement to reflect on. Researchers, in particular, felt it was necessary to reflect on the power they have regarding what gets reported on in papers. For instance:

‘Recognizing that I have a lot of power in that scenario…Like, I could report whatever I want on engagement and not check in with [advisors], and honestly, people wouldn't really know. That's kind of the downside of reporting guidelines. […] You're still just taking someone's word for it, and the word is ultimately the researcher's.’ (P06‐R)

Lastly, several participants felt that research teams should consider reflecting on impact, including the value and benefit engagement has to the PWLE/F engaged.

### 3.3 Guidance Around Co‐Authorship With PWLE/F

PWLE/F and researchers provided guidance for enabling meaningful co‐authorship with PWLE/F on papers. Based on their experiences with co‐authorship, research teams should: (1) recognise and create opportunities for co‐authorship with PWLE/F; (2) have frequent meetings, check‐ins and writing sessions with PWLE/F and (3) have conversations around writing, co‐authorship and terminology with PWLE/F.

#### 3.3.1 Recognise and Create Opportunities for Co‐Authorship

Both PWLE/F and researchers felt it was critical to recognise and create opportunities for meaningful co‐authorship with PWLE/F. Co‐authorship was viewed as a way for PWLE/F to receive ‘the credit that they deserve’ (P11‐P) for their contributions. Moreover, participants felt that opportunities for co‐authorship should be meaningful and tailored to PWLE/F needs. For instance, one researcher stated, ‘It's not just sending them the final version of the manuscript and saying, “Do you agree with this?”’ (P18‐R). Others found asking PWLE/F about their goals to be helpful:

‘Asking [advisors] what they want to get out of this opportunity. Cause we don't know and maybe we can facilitate some of those things. “Are there things that you want? Are there any skills you want to develop?” I think those are also important when doing engagement. Maybe we're able to provide some professional development and training.’ (P13‐R)

Researchers felt that providing opportunities for training and mentorship for PWLE/F would minimise power differences, help PWLE/F make ‘a meaningful contribution’ (P08‐R) to the research, and ultimately, improve the co‐authorship process.

#### 3.3.2 Have Frequent Meetings, Check‐Ins, and Writing Sessions

Many participants found ongoing meetings, check‐ins and writing sessions beneficial to co‐producing a manuscript with PWLE/F. Both PWLE/F and researchers felt it was important to bring PWLE/F in as early on in the writing process as possible and set clear expectations ‘from the get‐go’ (P18‐R) to ensure everyone is on the same page. One participant noted:

‘You've had multiple opportunities to bring in people with lived experience—and not just at the end of a project for validation purposes.’ (P19‐P)

A few PWLE/F felt it was helpful when researchers prepared questions beforehand and provided clear feedback. Additionally, as research teams produced papers with an engagement component, participants emphasised how teams should also check in with PWLE/F throughout the writing process and ensure they are fine with what is reported in the papers.

#### 3.3.3 Have Conversations Around Writing, Co‐Authorship and Terminology

PWLE/F and researchers emphasised having conversations around writing roles, co‐authorship and terminology. A few researchers spoke about how it was important to have conversations with PWLE/F about how to ‘work together and write together’ (P07‐R) and the ‘pros and cons of being included versus not’ (P05‐R) regarding co‐authorship. PWLE/F expressed that research teams should provide clarity around where papers will be published, what information about them will be shared and who the paper is accessible to:

‘It's important to be very clear with people where this is gonna appear. Who may see it? […] People that aren't involved in research may not really have a clear understanding of who this will be accessible to, and whether it's going in publications.’ (P15‐F)

This included discussing terminology and label preferences with PWLE/F, as it will vary, in addition to providing accommodations that could help preserve anonymity. Importantly, PWLE/F should be given the power to decide how they are identified in papers:

‘[advisors] get to decide what are the pieces of the jigsaw puzzle they want to put out there. Because there are ways to piece together people's identities, specifically with social media and all the information that's out there.’ (P04‐P)

Engaging in these conversations early on was understood as a means of building trust and rapport between co‐authors, in addition to ensuring PWLE/F felt comfortable in their roles.

#### 3.3.4 Build Open and Honest Relationships With PWLE/F Co‐Authors

Relationship‐building was perceived as a fundamental part of meaningful co‐authorship. Both PWLE/F and researchers felt that building open and honest relationships with PWLE/F co‐authors helped improve the research team's experience. This included a ‘relationships first’ (P07‐R) approach, such as efforts on the researcher's side to create a space where co‐authors are treated as equals, feel valued and are kept in the loop.

### 3.4 Practical Tips for Reporting on Engagement

As researchers and PWLE/F reflected on their writing experience, many had similar tips for reporting on engagement in papers. This included: (1) loopholes when limited with word count; (2) including PWLE/F in the writing process and (3) specific language considerations for mental health and/or substance use.

#### 3.4.1 Loopholes When Limited With Word Count

Word limits set by journals were seen as a major barrier to reporting on engagement; however, PWLE/F and researchers found loopholes in navigating limited word counts. This included using tables and figures to describe engagement in their papers, adding the engagement plan to the appendix section, submitting to journals with higher word counts and writing a separate process paper for more detail. For example:

‘A simple table goes a really long way in terms of illuminating how you engage people and that would enhance accountability.’ (P19‐P)

#### 3.4.2 Asking PWLE/F to Take Part in the Writing Process

Some felt that including PWLE/F in the writing process, such as creating space for them to write in the paper, could help the engagement process:

‘There could be very easily a section where the person that usually takes the lead in writing the article asks for comments, leaves a blank space, and says, “Hey, can you please describe yourself here?” So then the person can contribute to the article, not only by reading the rest of it and providing feedback, but actually writing about themselves.’ (P18‐R)

#### 3.4.3 Language Considerations for Mental Health and/or Substance Use Research

In the context of mental health and/or substance use, participants raised specific considerations related to terminology and stigma. PWLE/F and researchers felt research teams should reflect on the terminology used in papers to describe PWLE/F and ensure there is a ‘shared framework for language’ (P20‐R) to prevent stigmatisation. Lastly, one researcher reflected on how language is constantly changing and to stay up to date with what is considered stigmatising:

‘People don't really use the word “addiction” anymore. There's so much stigma already associated with it. You want to be careful how you report. Most journals will catch it, but some people are just behind.’ (P13‐R)

### 3.5 Considerations for Journals

As a higher level, several participants felt that ‘journals need to change their standards’ (P18‐R) and highlighted specific considerations for journals to facilitate both the meaningful reporting of engagement and co‐authorship with PWLE/F. These included: (1) increasing journal word counts for authors to write about the engagement process and impact; (2) a general need for journals to change to be more engagement‐centric; (3) flexibility around pseudonyms and anonymity for PWLE/F co‐authors; and (4) creating opportunities for PWLE/F to publish on their own.

## 4 Discussion

### 4.1 Overview

The present study aimed to explore what PWLE/F partners and researchers perceived are important facets of reporting on engagement in mental health and/or substance use research. To address current limitations with reporting, participants identified a need for tailored guidance that is flexible and reflective yet can promote transparency, accountability and learning in the field. We, therefore, developed a reporting guidance document for engagement in mental health and/or substance use research that balances flexibility and standardisation while incorporating reflection into purposeful reporting. The guidance document is intended to be used as a roadmap—not a checklist—to help guide authors to meaningfully write about engagement without the rigid boundaries of a reporting guideline. Guidance around co‐authorship with PWLE/F partners was also included.

### 4.2 Reporting Guidelines: A Checklist or a Roadmap?

Although many journals require reporting guidelines, they are also heavily criticised—this is especially the case for qualitative research. Reporting guidelines in this discipline are critiqued for being overly prescriptive, reductionist and methodologically incongruent with qualitative research and are even rejected by several qualitative journals [21, 27, 28]. A similar critique is seen with reporting guidelines for patient engagement, notably the GRIPP, as promoting a false sense of objectivity, ignoring power imbalances and not encouraging reflexive reporting [15]

However, the utility of guidelines cannot be ignored. Guidelines can improve transparency in research and help authors new to a field with reporting in a way that is methodologically congruent to their research [21]. While frameworks such as the GRIPP can provide general reporting guidance for engagement, they might not capture the nuances of engagement in mental health and/or substance use research, such as how it is shaped by stigma, power imbalances and disclosure concerns. As engagement is inadequately reported in mental health and/or substance use research [3, 6, 29], reporting guidance is needed such that authors have the tools to better report on engagement in their published work.

Our findings show that while guidelines provide a foundation, it is the flexibility and reflexivity within them that will allow engagement to be communicated authentically. Our goal here is not to stifle the use of strong, evidence‐based guidelines such as the GRIPP. We only intend to provide an alternative, reflective guidance that is rooted in the experiences of researchers and PWLE/F in mental health and/or substance use research. Similar to how reporting guidelines can help with methodological congruency, we hope that our guidance document will help with experiential congruency—that is, a means of helping research teams align engagement practices with reporting without imposing rigid standards.

### 4.3 Co‐Authorship Guidance: What Is New and What Is Next?

The International Committee of Medical Journal Editors (ICMJE) criteria for co‐authorship defines authorship based on: (1) contributions to conception, design, data acquisition, data analysis or data interpretation; (2) drafting or revising the work; (3) approving the final version and (4) agreeing to be accountable for the work [30]. They also state that ‘all individuals who meet the first criterion should have the opportunity to participate in the review, drafting, and final approval of the manuscript.’ [30]. Therefore, if PWLE/F partners have contributed to the study conception, design, acquisition, analysis or data interpretation, we should be creating and presenting them with the opportunity for co‐authorship. However, as the ICMJE does not provide specific recommendations for co‐authorship with PWLE/F, there has been a call for revisions or expanded guidance on how to apply the ICMJE guidance to PWLE/F authorship [31].

Although there is limited guidance for co‐authorship with PWLE/F in mental health and/or substance use research, guidance exists for patient authorship in health research [32, 33]. For instance, a systematic review by Arnstein et al. [32] and a commentary by Richards et al. [33] provide guidance for patient co‐authorship in health research. Arnstein et al. [32] outlines relevant recommendations for involving patient authors throughout the stages of manuscript preparation that reflect our findings (e.g., early involvement, clarifying roles and providing ongoing updates). Richards et al. [33] state the importance of ensuring a common understanding of the publishing process between ‘patient’ co‐authors and researchers. They further provide an overview of the publication process and common terms to be shared with patient co‐authors [33].

Research teams across health disciplines would benefit from these guidances; however, there are specific considerations unique to mental health and/or substance use research that are overlooked. Notably, our findings showcase the importance of having discussions around labels for PWLE/F and the importance of flexible accommodations, given the risks of potentially stigmatising labels. These are areas that are not discussed in guidances for co‐authorship in health research. For example, Arnstein et al. [32] recommends identifying in the manuscript which authors are patients, yet the risk of ‘outing’ PWLE/F in mental health and/or substance research should be an informed decision made by the PWLE/F engaged. For example, in our findings, some PWLE/F felt there were potential personal and professional risks with disclosure, highlighting the need for flexible authorship options.

Based on our findings, we recommend completing the reporting with PWLE/F co‐authors and offering them opportunities to write a piece around their experience with engagement in papers if this is within their interests and skillset. While this added step might introduce complexity—including additional time and reflection for the research team—it can ultimately enhance the depth and authenticity of what is reported. We acknowledge that depending on the subject matter, co‐writing sections with PWLE/F might not be as feasible. However, creating opportunities for writing in areas that might be more impactful for PWLE/F, such as reporting on engagement processes and their experiences with engagement, is a valuable step forward in co‐producing academic content. These opportunities can also be beneficial for PWLE/F—in particular, youth and younger adults—and help with professional development [18, 34].

### 4.4 Recommendations for Journals

Our study reveals several areas for journals to consider with regard to publishing engagement‐related work. Journal restrictions on word count have previously been described as a major limitation to sufficiently reporting on patient engagement in health research [35, 36, 37]. We recommend that journals consider increasing the word count for engagement‐related papers so that authors can accurately describe engagement activities and impact in‐depth, ultimately promoting transparent research. Moreover, in light of the calls to identify PWLE/F engaged on publications by listing their authorship credentials as ‘Patient Author’ [38], we recommend that journals have flexibility around credentials that are catered to the needs and comfort levels of the PWLE/F engaged, in addition to highlighting options for anonymity such as pseudonyms. Lastly, we recommend that relevant journals (e.g., psychiatry journals and engagement journals) promote reporting guidance as suggestions (not requirements) to foster good reporting and transparency in engagement‐related research.

### 4.5 Strengths and Limitations

This study has several strengths, such as including PWLE/F engagement throughout the study, recruiting a relatively diverse sample across Canada and developing a guidance document rooted in the experiences of PWLE/F and researchers. However, there are several limitations. This is a first draft of a guidance document developed from interviews with PWLE/F and researchers and refined through meetings with PWLE/F and researchers on the research team. The transferability of the findings in a different context, such as outside of Canada, should be considered [39]. Although, in theory, the guidance document can be used by any engagement‐focused research team, transferability outside of mental health and/or substance use research might be limited and should be explored. Moreover, future research should consider further exploring the relevance of the guidance document with a larger sample of Indigenous peoples, racialized populations, newcomers and other specific sub‐populations involved in engagement work. Indeed, as recruitment primarily occurred through our professional network based out of an academic university and teaching hospital, it is possible that PWLE/F participants with primarily positive research experiences were self‐selected. Lastly, recruitment relied entirely on self‐reported data to confirm participants' experience with co‐authorship, acknowledgements and research experience, without external verification.

## 5 Conclusion

As engagement is inadequately reported on in mental health and/or substance use research, we sought to develop an evidence‐based reporting guidance document rooted in the experiences of PWLE/F and researchers. The guidance document follows a structured, yet flexible approach to reporting that avoids a rigid checkbox approach and prioritises reflection. We encourage research teams that engage PWLE/F to consider using the guidance document as they write up their work. As the guidance document is intended to be a living document, future research should focus on further evolving the document to capture a wider international audience across different contexts outside of Canada. Ultimately, in a climate where meaningful engagement is increasingly prioritised but guidance on how to achieve it remains limited, this guidance document can be used as a resource to advance the field by providing a roadmap to more transparent, consistent and reflective engagement work.

## Author Contributions

**Natasha Y. Sheikhan:** conceptualisation, data curation, formal analysis, writing – original draft, methodology, project administration, writing – review and editing, software, funding acquisition, resources. **Kerry Kuluski:** conceptualisation, writing – review and editing, supervision, methodology, funding acquisition, resources, formal analysis. **Melissa Hiebert:** conceptualisation, formal analysis, methodology, writing – review and editing, resources. **Charlotte Munro:** formal analysis, writing – review and editing, methodology. **Vivien Cappe:** formal analysis, methodology, writing – review and editing. **Mary Rose Kesteren:** formal analysis, methodology, writing – review and editing. **Sean Kidd:** conceptualisation, writing – review and editing, methodology, formal analysis. **Lisa D. Hawke:** conceptualisation, formal analysis, writing – review and editing, supervision, methodology, funding acquisition, resources.

## Ethics Statement

Our study was approved by the University of Toronto Research Ethics Committee (RIS human protocol no. 45931) and approved for recruitment by the Centre for Addiction and Mental Health Research Ethics Board (no. 2024/071). Before participating in the study, all participants provided written informed consent via the REDCap software.

## Conflicts of Interest

The authors declare no conflicts of interest.

## Supporting information

Supporting information.

## Data Availability

The data that support the findings of this study are available from the corresponding author upon reasonable request.
