# Supplementary material for: Co‐Authoring and Reporting on Lived Experience Engagement in Mental Health and/or Substance Research: A Qualitative Study and Guidance Document
Source: Health Expect. 2025 Mar 12;28(2):e70198. doi: 10.1111/hex.70198 (PMC11898218; doi:10.1111/hex.70198)
Supplement: Supplementary file 1 — Supporting information. [file HEX-28-e70198-s001.pdf]

camh
mental health is health

Institute of Health Policy, Management & Evaluation
UNIVERSITY OF TORONTO

CIHR IRSC
Canadian Institutes of Health Research
Instituts de recherche en santé du Canada

Reporting on lived experience engagement in research papers

A guidance document tailored to engaging with people with lived experience and families in mental health and substance use research

Created by

Natasha Y. Sheikhan, Kerry H. Kuluski, Mel Hiebert, Vivien Cappe, Charlotte Munro, Mary Rose van Kesteren, Sean Kidd, and Lisa D. Hawke

Last updated on

10/12/2024

01 OVERVIEW

Objectives

1. To promote transparency and accountability regarding people with lived and living experience and families (PWLE/F) engagement in mental health and/or substance use research.
2. To balance flexibility and standardization when reporting on how PWLE/F are engaged.
3. To help authors report on PWLE/F engagement in papers in a reflective manner.

Definitions

People with lived and living experience and families (PWLE/F): People who identify as having past or ongoing personal, family, or caregiver experiences of mental health and/or substance use. They are increasingly included as partners in research studies (also referred to as PWLE/F partners).

PWLE/F engagement: The process by which PWLE/F are included as partners or advisors in research studies, rather than as participants.

Peer-reviewed publications: Papers produced by research teams and submitted to academic journals for a process called 'peer review'.

Scope

This guide is flexible and not intended as a prescriptive checklist, recognizing the uniqueness of each engagement effort. We hope to help authors see new pathways to success when writing about PWLE/F engagement in research papers.

The guide is anchored in the following core values:

Value 1
Transparency about the engagement process

Value 2
Flexibility with what is reported and how it is reported

Value 3
Reflection as a key part of the reporting process

Audience

The intended audience is research teams (e.g., researchers, PWLE/F partners, research organizations) conducting engagement in mental health and/or substance use research. This includes people who are both experienced and new to engagement. Although the guide was developed in the context of mental health and substance use, it may apply to other fields of health research, especially in research areas where stigma and power are prominent.

How was the guide created?

The guide was created as part of a PhD project at the University of Toronto and Centre for Addiction and Mental Health. It was developed through:

- Virtual interviews with 13 PWLE/F and 12 researchers across Canada; and
- Refined through feedback from PWLE/F partners and researchers on the PhD project

The PWLE/F interviewed had : (a) experience being engaged as a PWLE/F partner or advisor in mental health and/or substance use research and (b) contributed to a journal article as a co-author or in an acknowledgement. The researchers interviewed had: (a) conducted research in the field of mental health and/or substance use; (b) engaged PWLE/F in research; and (c) reported on engagement in journal articles.

How should teams use it?

This guide should be used as a tool for reflection, helping people think through areas to report on that are relevant to their project. We recommend that researchers and PWLE/F partners review the document together so that PWLE/F contributions are told from their perspectives. This approach can help address power dynamics in deciding what gets reported in papers, ensuring that all perspectives are represented.

Researchers and PWLE/F are encouraged to reflect individually and as a team, bringing their insights to team discussions. **This guide should be used as a roadmap, not a rigid checklist.**

02 REPORTING FRAMEWORK

Key areas for reporting and reflection

The following table highlights key areas for reporting and reflecting on engagement in your papers. It includes a framework for what to report on, guiding questions, and additional reflection points. **We recommend that authors disclose who completed the table in the paper to enhance transparency.**

Areas to report and reflect on	Guiding questions	Additional reflections
**Who** was involved?	• Who were the PWLE/F engaged? Are there any descriptive factors that are relevant to the research question?<br><br>Note: If describing PWLE/F, consider also describing researchers.	For PWLE/F partners, consider reflecting on how you want to be referenced in papers.
**What** were the activities, roles, and responsibilities?	• What were the specific activities for engagement?<br>• What were the roles and responsibilities of the PWLE/F partners?<br>• What was the level or extent of engagement throughout the project?<br>• Was there any training provided for the PWLE/F engaged?	Consider reflecting on your social positioning and the role you have on the research team, in particular, the roles of co-authors.

**How** did you go about the engagement process?	• What did the engagement process look like? What were the decision-making processes and feedback loops?<br>• How were relationships built? Was there a terms of reference?<br>• What was the engagement context (*e.g.*, virtual, in person)?<br>• Did you compensate your PWLE/F partners?<br>• How did you recruit PWLE/F partners?<br>• Were there any accommodations provided to make the space more inclusive?<br>• Are there any influencing factors that facilitated the engagement process? Any factors that acted as barriers? What were the solutions and lessons learned?<br>• How were power imbalances addressed?	Consider reflecting on why you are engaging PWLE/F in the first place, how power appeared during the engagement process, and any efforts the team made to minimize the power dynamic between researchers and PWLE/F partners.
**When** did engagement occur?	• What did the timeline for engagement look like?<br>• At what stage(s) of the research process were PWLE/F engaged?<br>• What was the frequency of meetings?	Consider reflecting on the timeline for engagement early on and if sufficient time was set for engagement activities.
**What** was the impact?	• What was the impact of engagement on the research project (*e.g.*, recruitment, analysis)?<br>• What was the impact on PWLE/F?<br>• What was the impact on researchers?<br>• Was engagement evaluated?<br>• What were the experiences of PWLE/F?	Consider reflecting on the value engagement has to the PWLE/F engaged. Was the experience good for PWLE/F? How did it benefit them? Were there any negative impacts?

Reporting Tip

Limited word counts in journals can be a barrier when describing your engagement process in papers. When limited with word count, consider:

1. Describing engagement through **tables, figures, or appendices.**
2. Writing a separate **process paper** to capture greater detail on the engagement process. You can cite this process paper in the methods section of future publications.
3. Submitting to **journals with higher word counts**, such as engagement-focused journals.

03 CO-AUTHORSHIP GUIDANCE

Recognize & create opportunities for co-authorship with PWLE/F.

Consider creating meaningful opportunities for co-authorship tailored to the **skills and needs of PWLE/F**. Ask PWLE/F what roles they might want to take part in or what would be valuable for their **personal and professional growth**. Note that **many tasks qualify people for co-authorship beyond drafting the paper**, such as contributing to study conceptualization, design, data collection, analysis, and interpretation of data. If PWLE/F are involved in any of these tasks, we recommend providing them with the opportunity to be a co-author, and if interested, sending them a draft of the paper.

Ensure early engagement.

If feasible, consider bringing PWLE/F co-authors in **early** during the writing process so that they can meaningfully contribute as co-authors and have a larger influence on the work.

Keep PWLE/F in the loop.

Maintain open communication by having **frequent meetings**, **check-ins**, and potentially **organizing writing sessions** if appropriate for your project. It might be helpful to prepare questions before meetings and provide ongoing feedback to PWLE/F co-authors. **Regularly update PWLE/F co-authors**, such as by sending them updates or drafts via email, to ensure they are comfortable with what is reported in the paper throughout the process.

Discuss roles & labels with PWLE/F.

Have conversations around **writing**, **co-authorship**, and **terminology** early on (*e.g.*, what does it mean to be a co-author?). Ensure PWLE/F are comfortable with the terminology in the paper, the labels used, and how they are being identified as co-authors (*e.g.*, are they identified as a PWLE/F on the paper?). **Provide options** for them regarding labels that suit their preferences (e.g., research assistant, PWLE/F partner). If needed, provide accommodations like anonymity. Understand PWLE/F holistic identity.

Be mindful of language considerations, especially for mental health and substance use research. Make sure you use person-centred and non-stigmatizing language and that the PWLE/F engaged are comfortable with the language used in the paper being produced.

Have support available.

If possible, provide opportunities for **training and mentorship** to help facilitate the co-authorship process. This can better equip PWLE/F to meaningfully participate as co-authors and can also help with their personal and/or professional goals.

Build relationships.

Build **open and honest relationships** with PWLE/F co-authors to enhance the experience for the entire research team. This might include efforts on the researcher's side to create a space where co-authors are treated as equals, feel valued, and are kept in the loop.

Acknowledgements

We would like to thank the PWLE/F and researcher participants interviewed for this project—we are incredibly grateful for your insights and your contributions are the foundation of this document.

This research is funded by the Canadian Institutes of Health Research (*award number: FBD-181375*) and the Ontario SPOR Support Unit Partnership Capacity Building Fund.
